# Implementation difficulties and solutions for a smart-clothes assisted home nursing care program for older adults with dementia or recovering from hip fracture

**DOI:** 10.1186/s12911-024-02468-5

**Published:** 2024-03-12

**Authors:** Chung-Chih Lin, Ching-Tzu Yang, Pei-Ling Su, Jung-Ling Hsu, Yea-Ing L. Shyu, Wen-Chuin Hsu

**Affiliations:** 1 Department of Computer Science and Information Engineering, College of Engineering, Chang Gung University, Taoyuan, Taiwan (R.O.C.); 2 School of Nursing, College of Medicine, Chang Gung University, Taoyuan, Taiwan (R.O.C.); 3 Department of Neurology, Linkou Chang Gung Memorial Hospital, Taoyuan, Taiwan (R.O.C.); 4 College of Medicine, Chang Gung University, Taoyuan, Taiwan (R.O.C.); 5 Dementia Center, Department of Neurology, Linkou Chang Gung Memorial Hospital, Taoyuan, Taiwan (R.O.C.); 6 Department of Nursing, Kaohsiung Chang Gung Memorial Hospital, Kaohsiung, Taiwan (R.O.C.); 7 Department of Gerontology and Health Care Management, Chang Gung University of Science and Technology, Taoyuan, Taiwan (R.O.C.); 8 Healthy Aging Research Center, Chang Gung University, Taoyuan, Taiwan (R.O.C.)

**Keywords:** Smart care, Dementia, Hip fracture, Home health, Smart clothing, Wearable device, Remote monitoring

## Abstract

**Background:**

Wearable devices have the advantage of always being with individuals, enabling easy detection of their movements. Smart clothing can provide feedback to family caregivers of older adults with disabilities who require in-home care.

**Methods:**

This study describes the process of setting up a smart technology-assisted (STA) home-nursing care program, the difficulties encountered, and strategies applied to improve the program. The STA program utilized a smart-vest, designed specifically for older persons with dementia or recovering from hip-fracture surgery. The smart-vest facilitated nurses’ and family caregivers’ detection of a care receiver’s movements via a remote-monitoring system. Movements included getting up at night, time spent in the bathroom, duration of daytime immobility, leaving the house, and daily activity. Twelve caregivers of older adults and their care receiver participated; care receivers included persons recovering from hip fracture (*n* = 5) and persons living with dementia (*n* = 7). Data about installation of the individual STA in-home systems, monitoring, and technical difficulties encountered were obtained from researchers’ reports. Qualitative data about the caregivers’ and care receivers’ use of the system were obtained from homecare nurses’ reports, which were explored with thematic analysis.

**Results:**

Compiled reports from the research team identified three areas of difficulty with the system: incompatibility with the home environment, which caused extra hours of manpower and added to the cost of set-up and maintenance; interruptions in data transmissions, due to system malfunctions; and inaccuracies in data transmissions, due to sensors on the smart-vest. These difficulties contributed to frustration experienced by caregivers and care receivers.

**Conclusions:**

The difficulties encountered impeded implementation of the STA home nursing care. Each of these difficulties had their own unique problems and strategies to resolve them. Our findings can provide a reference for future implementation of similar smart-home systems, which could facilitate ease-of-use for family caregivers.

## Background

Taiwan has improved the availability of health care for persons with chronic diseases who are home-bound or living in rural communities by providing remote health care through telehealth. Remote care via telehealth care has improved care for persons with metabolic syndrome living in a rural area [1] as well as facilitating in-home control of hypertension [2]. In a Taiwan-based qualitative study, telehealth care was provided to family caregivers of patients with heart failure following hospital discharge [3]. When compared with caregivers who only received discharge planning, those who received home telehealth support experienced reduced caregiver burden, and mastery of stress and family function improved. during the first 30 days after discharge of persons with heart failure for disease management Another qualitative study explored responses of users of home telehealth care for chronic disease management [4]. Most users perceived telehealth care as a convenient and useful model for healthcare delivery.

Smart home care technologies that monitor behaviors of persons needing assisted care are typically equipped with interrelated software and hardware components. The goal is to collect data that can alert their caregivers or health care professionals about activities that might be risky so that they can take preventive actions [5]. This merging of smart technologies and the Internet of things (IoT) was used by Amiribesheli and Bouchachia (2018) to develop a smart technology-assisted (STA) homecare prototype tailored to the needs of persons living with dementia (PLWD) [6]. To determine if the prototype could reduce dementia care difficulties, the prototype was evaluated with input from dementia-care stakeholders comprised of PLWD, caregivers (formal and informal), and geriatric psychologists. The findings of Amiribesheli and Bouchachia suggest that STA care could be used for family caregivers of older relatives living at home with chronic care needs, such as older adults recovering from hip-fracture surgery or PLWD.

Hip fracture is a common traumatic injury for older adults, which is associated with morbidity and mortality. Hip fracture is increasing as the population ages and recovery from this injury is a major social and economic burden, partially due to the cost of care [7, 8]. The risk of osteoporotic hip fractures for individuals ≥ 50 years was estimated to be about 158 million in 2010 and is expected to double by 2040 [8]. It is estimated that by 2050, Asia will account for half of all hip fractures [9].

The increase in older adults worldwide has also contributed to an increase the number of persons living with dementia. This health issue is another cause for the increasing cost of health care for societies worldwide [10]. The World Alzheimer Report from 2015 estimated that the number of persons living with dementia would double every 20 years, increasing from 46.8 million to 131.5 million by 2050 [10]. In response to this expected increase, the World Health Organization announced a global public-health action plan for 2017 to 2025 designed to increase care for persons living with dementia and their families and decrease the impact of dementia on communities and countries [11]. In 2014 in Taiwan, all-cause dementia was estimated to have an age-adjusted prevalence of 8.04% [12]. The proportion of adults ≥ 65 years in Taiwan was 14.6% in 2018; in 2026 it will exceed 20% [13]. Therefore, improving home care for older persons recovering from hip-fracture surgery and those living with dementia is an important health and societal issue.

In Taiwan, family caregivers provide long-term in-home care for over 80% of older adults recovering from hip fracture and over 90% of PLWD [14, 15]. An STA home nursing care program could benefit these family caregivers by providing home health care support, which could improve home care and reduce caregiver burden. Therefore, we developed an STA home nursing care program for family caregivers to alleviate some of the burden of caregiving and reduce the need for constant vigilance, which is required when providing care for older persons recovering from hip-fracture surgery or PLWD. We incorporated smart clothing technology developed originally by Wang et al. [16] for monitoring electrocardiography (ECG) signals. The smart clothing was also demonstrated to be accurate for variables for older adults living in long-term care institutions in Taiwan [17] and has been used to alert persons to arrythmias that occur during exercise [18].

This study describes installation of the STA home nursing care program, modifications to a smart vest, which incorporated smart technology designed specifically for the older persons recovering from hip-fracture surgery or living with dementia, and measures monitored. Reports from engineers and nurse researchers were used to describe problems encountered during installation and implementation of individual in-home systems, and the strategies used to resolve them. Analysis of feedback collected by home care nurses identified difficulties caregivers experienced with the system.

## Methods

### Design of the smart vest

**Fig. 1:**
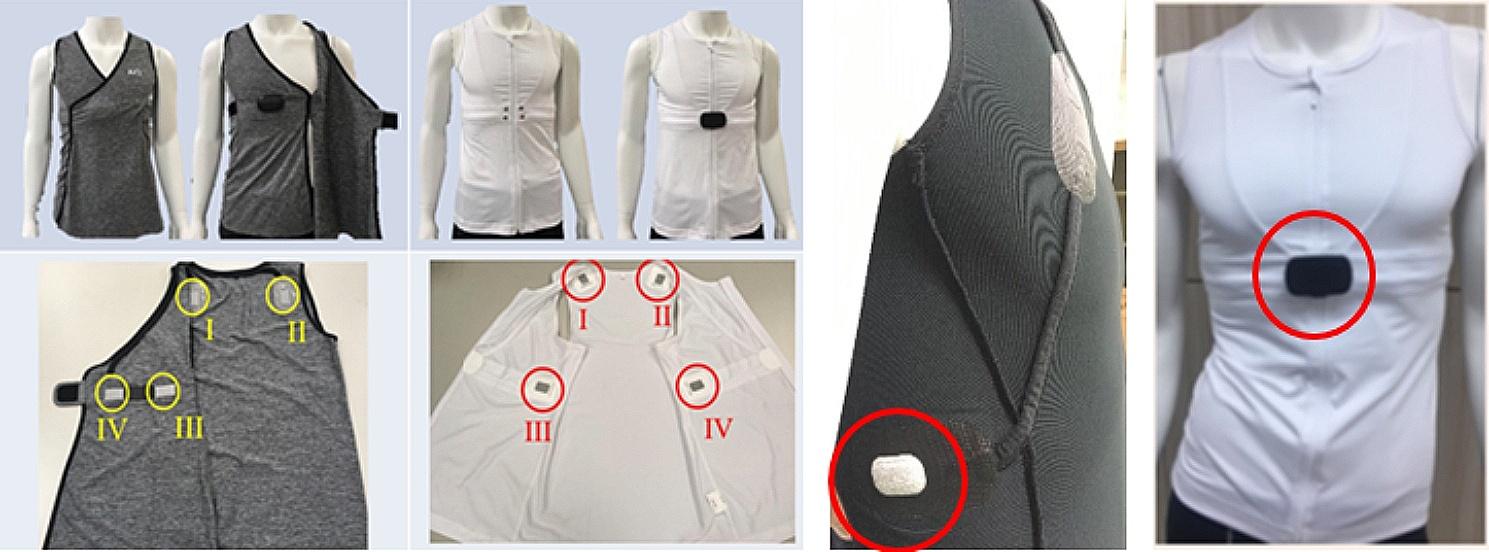
Views of the smart vest with the four locations for the sensors: cervical spine, upper abdomen, body mass center, and both sides of the waist (I, II, III, and IV, respectively) The images on the far right show the conductive knitted fabric containing the buckle used to secure the vest and complete the circuit

The smart vest was made of a washable electroconductive fabric with sewn-in electrode patches that collect analog signals from the four sensors; the circuit is completed by fastening a metal buckle (Fig. 1). The prototype for the smart vest platform integrates signals from multiple sensors. In this study, we only used a G-Sensor for gravitational acceleration (GC), which provided data about activity level, gait, and posture. Application of an algorithm based on the hidden Markov model (HMM) was used detect falls. The smart vest is worn as underwear and can accurately gauge acceleration of movement on three axes; it was modified to make it easy for older persons to wear and is off-the-shelf ready. The ECG sensor nodes are easy to remove from the smart-vest, allowing and the signal transmission metal fiber containing the ECG electrodes to be washed directly in the washing machine and line-dried. The durability of the fiber after washing has been tested at least 20 times. Every care receiver was provided with at least two vests to allow continued use when a vest needed washing.

**Fig. 2:**
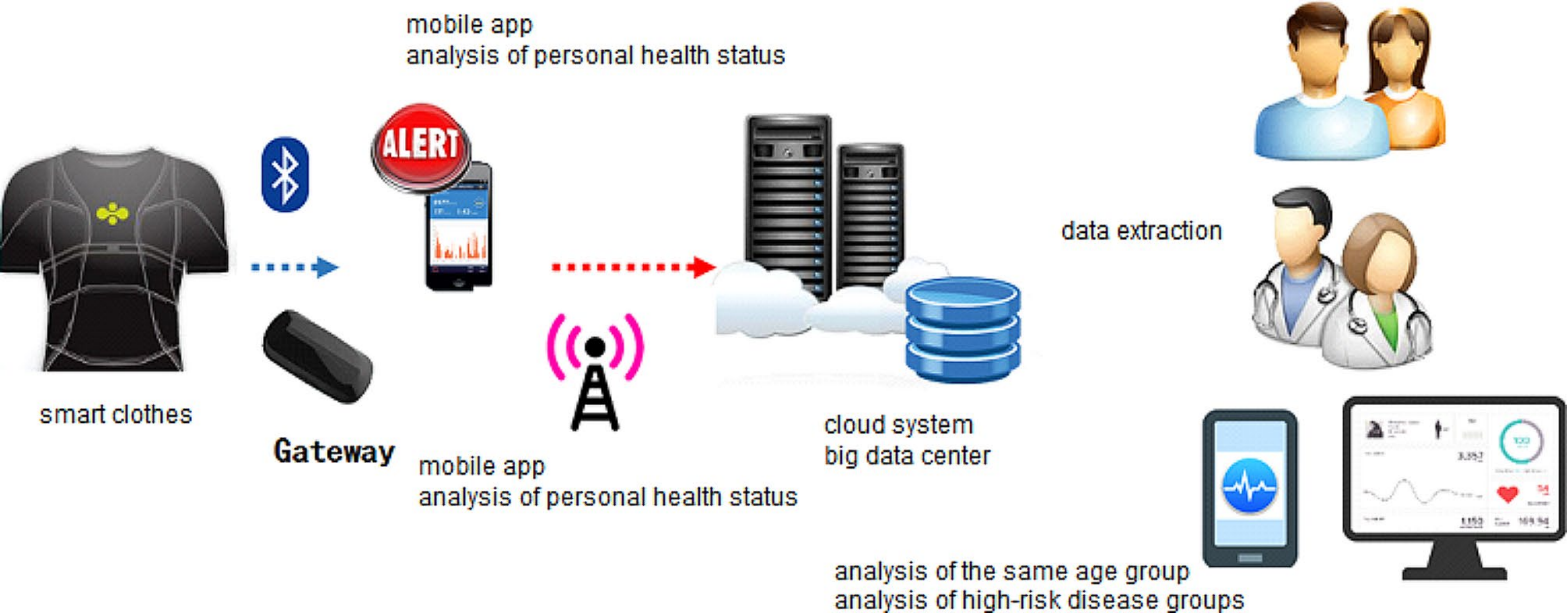
Illustration of the smart technology-assisted home health care system

While the smart vest is in place, signals are sent to a sensor node, which is an analog-to-digital converter (ADC) microprocessor for analysis in real time. The ADC converts digital signals with a sampling frequency of 250 Hz. A smartphone mobile application (APP) is used as a gateway server to display results of analysis from the sensors and a cloud platform integrates longitudinal health data from the smart vest, which can be used for healthcare consultations with nursing staff (Fig. 2). Family caregivers and the home-care nurses downloaded the APP to their mobile phones to receive health and emergency information. Details of the design and technical information have been previously reported [16]. These smart-clothing sensors and the electroconductive fabric have been awarded 25 patents and four technology transfers.

### The STA Home nursing-care program

The STA home care program was developed to be used in combination with the smart vest, described above. The heterogeneity among older PLWD and older adults recovering from hip-fracture surgery allowed us to develop a program that assessed daily living patterns for older adults with different disabilities.

#### Preparation and installation of the STA home care program

The first stage of preparation for the home care program was conducted by a research nurse who visited the home of each caregiver to assess the home environment. A floor map was drawn to indicate the position of the living room, bedrooms, and exit doors. The map was discussed with the engineer who then prepared the sensors and web environment. The research nurse made a second visit to the home to install the sensors in the living room and bedrooms; for caregivers of PLWD, a sensor with an audible alarm was placed by exit doors. A smoke detector was installed in all homes to signal potential fire and smoke. In addition, the homes were equipped with an emergency push-button to alert the home care nurse (via the APP) if emergent care was needed. Initially, the setup required six hours, which included installation of sensors and integration with the internet system. This lengthy setup was an inconvenience for other family members in the household. However, once standardized, installation required approximately three hours.

#### Settings specific to persons with dementia

Because wandering is one of the most significant concerns for caregivers of PLWD, indoor positioning sensors were installed in the door and in areas of frequent activity. A Received Signal Strength Indicator (RSSI) was used for indoor positioning was used because signals from a Global Positioning System (GPS) are frequently weak or unavailable in indoor environments. RSSI measures the power level of received wireless signals, such as Wi-Fi or Bluetooth, and can be used to estimate the distance between a mobile device and a set of fixed reference points. Accuracy was maintained through constant data collection and calibration, signal strength to distance mapping, fingerprinting, localization, error mitigation, and continuous refinement.

### Measurements

The STA home nursing care program used a remote monitoring system paired with the smart vest; sensors were placed in each participant’s home to facilitate detection of the care receiver’s activities. Daily steps and other movements were used to monitor level of daily activity, which included: (1) active steps; (2) gait, such as forward, backward, swinging; 3) posture status while lying, sitting, standing, walking; (4) getting out of bed; and (5) going to the toilet. Monitoring for PLWD also included level of intensity of the RSSI in the sensors, which were assessed every 30 s.

The smart-technology allows individual alert thresholds to be set for each person based on their disability. Analyzed data from the smart vest were transmitted and downloaded to an APP on the nurse’s mobile phone, which included alerts about any atypical behaviors, such as wandering or a lack of movement, which were then immediately communicated to family caregivers. Any modifications to caregiving activities that might mediate these behaviors were initiated immediately through the nurse, such as encouraging daily activities, providing safety measures for getting up at night or going out, and managing the agitation of persons with dementia.

Monitoring data were collected from sensors in the smart vests and the home environment. Care receivers were instructed to wear the smart vest for at least four days/week for 24 h for three months, a length of time shown to allow detection of frequent falls [19]. Data transmitted from the sensors in the smart vest to the APP and to the cloud were analyzed by the researchers. Following installation of the sensors in the home environment, a research nurse visited the participants’ homes once a week for one month. During each home visit, the research nurse routinely checked the function of the system. She also made phone calls to verify and record the reasons for system alarms.

### Participants

We recruited older PLWD or recovering from hip fracture and their caregivers to participate in the STA home nursing care program from the trauma wards of Chang Gung Memorial Hospital at Linkou or neurological clinics in Taipei and Taoyuan. Patients were included by these criteria: (1) ≥ 60 years old, (2) received hip arthroplasty or internal fixation or were diagnosed with dementia by a neurologist, (3) lived in a home setting (4) lived in northern Taiwan, and (5) could communicate. Patients were excluded by these criteria: (1) terminally ill, (2) without a primary family caregiver, or (3) without a fixed home/living environment (living in an institution or rotating habitation). Family caregivers were included by these criteria: (1) ≥ 20 years old, (2) responsible for providing direct care or supervising care received by the patient, and (3) lived with the person with dementia or hip-fractured person. Potential participants were contacted at hospital admission or in clinics by a research nurse. Those who agreed to participate signed an informed consent.

Of 196 patients who were informed of the study, 107 were recovering from hip fracture and 89 had dementia; 179 patients refused to participate for the following reasons: the family caregiver did not feel it was necessary (*n* = 38), the patient did not want to wear the smart-vest or found it uncomfortable (too hot; *n* = 23); caregivers felt bothered by setting up the smart-care environment (*n* = 32); caregivers did not want to have another internet system in the house (*n* = 10); and caregivers felt they had to wait too long for the system to be set up (*n* = 4). Six patients agreed to participate but did not receive the smart-care intervention due to mortality (*n* = 1), moving to an institutional setting (*n* = 1), changed their decision due to fear of the potential harms of electromagnetic waves (*n* = 1), and difficulty in installing an internet web system at home (*n* = 3). The remaining patients with dementia (*n* = 7) and hip fracture (*n* = 6) agreed to participate and provided informed consent. However, one participant with hip fracture (S005) refused to use the smart-care system after agreeing to participate due to disruptions in the family while we attempted to install the home system. Therefore, this study reports the data for 12 participants who received smart-care, seven with dementia and five with hip fracture. Four participants with dementia were male; four had been diagnosed with very mild dementia, two with mild dementia, and one with moderate dementia. Of the five participants with hip fracture, four were female; all five were recovering from hip-fracture surgery.

### Data collection

The research team and engineers met monthly to share and discuss problems encountered during installation and maintenance of the home system. Research nurses maintained a detailed record of each home visit, which included documentation of the problems participants had encountered using the system. Problems that were ongoing as well as those that had been resolved were recorded. Home care nurses documented feedback from family caregivers about challenges with the use of the smart vest and home sensors. Data for feedback from the caregivers were analyzed with thematic analysis to match the difficulties with the implementation problems.

### Data analysis

Reports from home care nurses about the caregivers’ experiences with the home system were analyzed with thematic analysis [20], which aims to identify patterns in the data to form emerging themes [21]. Data describing problems experienced were coded and analyzed according to group difficulties and problems with implementation. The coders included an experienced qualitative researcher and two geriatric nursing specialists. Data were initially coded independently, and the two researchers then compared findings until a consensus was reached. After notes obtained from the home nurse about the experiences of the 12th participant were analyzed, the two researchers noted that no new information or insights had been identified, and both agreed that saturation had been reached with the 11th participant [22].

### Trustworthiness

Trustworthiness of the analysis of the reports was ensured by four criteria for rigor: credibility, dependability, transferability, and confirmability [23]. Credibility was enhanced by triangulation of data collected during home visits with the records of the nurse researchers home visits. Dependability and confirmability was enhanced by maintaining an audit trail of the data collection and analysis, and regular reviews of raw data, codes, and emergent themes, which were conducted by an outside panel of experts in gerontological nursing and qualitative studies. Transferability, which refers to the application of the findings, was enhanced through thorough rich descriptions and contextual conceptualizations of the study results.

## Results

Reports from the nurse researchers indicated three areas served as barriers to implementation: incompatibility with the home environment, errors in data transmissions, and inaccuracies in the data source. Field notes from the research team described the problems and what strategies were employed to attempt to mitigate these barriers to the smart-care system. The problems and solutions employed to address these difficulties are summarized in Table 1. Details are described below.

**Table 1:** Problems and solutions for the three barriers to implementation the home smart-care system

Barrier	Problem	Solution
**Incompatibility**	Internet set-up	Installation of service for those without a provider Application for a fixed IP address Stable telephone carrier for those needing a mobile hotspot
	Unstable internet system	Change of carrier Re-set of IP address
	Dysfunctional sensors	Switched from wireless to hard-wired
**Transmission errors**	Sensor accuracy	Reset sensor parameters for hip-fracture patients
	Battery durability	Installed longer-life batteries Timely battery changes Disconnected sensors not in use
	Loss of electrical power	Remotely rebooted system; Rechecked the system
	Accidental disconnection	Visited home to determine cause; rebooted the system
	Smart vest not connected	Nurse researcher alerted caregivers
	Smartphone APP error	Engineer reconnected
**Data inaccuracies**	Alarm thresholds	
	Persons with hip fracture	Created a formula to reset threshold during recovery
	Persons with dementia	Adjusted threshold to indicate levels of high and low activity
	Adherence to wearing vest	Explained importance of wearing the smart-vest to provide 24-h data
	Improper use of alarms	
	Emergency button	Emphasized the importance of using the button for emergencies only
	Door alarm	Despite explaining the importance of the sensor, this was not resolved.

*Incompatibility* with the home environment; *Transmission*, errors in data *transmission*; *Data inaccuracies, inaccuracies* in the data source

### Incompatibility

Incompatibility of the system differed for each home environment. However, one or more of the following variables was a contributing factor, which required adjustments specific to each setting: lack of an internet system, an unstable internet system, dysfunctional wireless sensors, or incompatible location of sensors.

#### Internet set-up

Six participants (S003, S004, S008, T002, T006, T008) did not have a home internet provider (46.1%) when the study began. In addition, homes with an internet provider needed a fixed Internet Protocol (IP) address under privacy protection regulations, which required an application to the provider. Although the engineer facilitated installment of an internet system where possible, and contacted the phone company on behalf of the family caregiver to explain why a fixed IP address was needed, resolving these problems required additional time from the engineer. In some instances (*n* = 3) it was not possible obtain an IP and a mobile hotspot was needed, which required the engineer to find a telephone carrier that was more reliable.

#### Unstable internet system

When the quality of the carrier resulted in instability of the home internet systems, we contracted with a more reliable commercial carrier or switched from a community provider to an independent provider. When the provider was a community service and the internet was unstable, we switched the family to an independent internet provider. In four instances (participants S001, S003, T001,T002), three months after beginning the study, the home monitoring system failed due to a sudden loss of the fixed IP. Two of these participants (S001, S003) also experienced problems with the smart-home monitoring system due to interference from other home utilities, and the fixed IP was re-set. Resolving these problems required extra visits from the engineer and research nurse, resulting in additional time.

#### Dysfunctional wireless sensor

Although the system was originally designed for wireless sensors to receive information from the smart vest, wireless transmission was blocked by the concrete walls in the home residence. This unforeseen difficulty was overcome by switching to hard-wired sensors.

### Errors in data transmission

Errors in data transmission were the result of sensor accuracy, battery durability, and loss of electrical power. These errors included inaccuracies in data as well as a loss of data due to interruptions in transmission.

#### Sensor accuracy

Sensor accuracy was a problem for caregivers of persons recovering from hip-fracture surgery. If the person with hip fracture used a walker or a cane, the sensors underestimated the number of steps; this resulted in an inaccurate assessment of changes in walking ability during recovery. This occurred for two participants (S004, S008) whose sensors did not detect any improvements in walking ability; a nurse researcher noticed the number of steps detected by the sensor and the actual steps taken was significantly different. It was determined that the small gait of these participants prevented sensors from obtaining an accurate count of steps. The parameters of the sensors were reset to accommodate the participants’ individual gaits, which resolved the problem.

#### Battery durability

Transmission was interrupted for four care receivers (participants S003, S007, S008, T008) because of battery failure. We solved this problem by purchasing a battery brand demonstrated in our initial monitoring to have the longest durability, changing the battery regularly in advance, and closing sensors for functions that were not currently being monitored.

#### Electrical power

Data for 12 participants was impacted by interruptions in or loss of electrical power. Data were lost for three participants (S001, S003, S005) when the electricity was accidentally turned off by a family member. Unfortunately, the loss of electricity caused a disconnection from the data center during a period of data transmission. Three participants (T002, S003, S004) were disconnected from the system when they removed their old vest, put on the second vest, and failed to attach the buckle correctly, which prevented completion of the circuit. The nurse researcher contacted the caregivers and instructed them to check the smart vest. For four other participants (S005, S008, T003, T006), data were not transmitted properly because the APP on the research nurse’s smartphone malfunctioned. The engineer resolved this problem by identifying and correcting the problem, re-establishing a connection with the network, and verifying that the system was functioning properly.

### Inaccuracies in the data source

The initial settings for the source of the data (the sensors) did not always align with real-life behaviors of the caregivers and care receivers. The settings for alarm thresholds, adherence to wearing the smart vest, and improper use of emergency alarms resulted in data that did not reflect the activity of the participants.

#### Alarm thresholds

The alarm thresholds needed to meet the needs of the care receiver and their caregiver, which differed for persons with hip-fracture and PLWD. The initial settings for persons recovering from hip-fracture surgery were based on their health status on hospital discharge. Because the rate of improvement varied with the measurement as well as with each individual, some guidelines were needed to adjust the settings during recovery. To better estimate the settings for low levels (initial status) to high levels (recovery phases), the nurse researcher determined the threshold for activity level could be calculated using the mean of the average daily steps during the first week of home care, plus and minus one standard deviation (SD). The threshold was calculated the week before entering the home care program. During the recovery phase, as the activity level improved over time, the threshold was readjusted monthly. For PLWD, it was critical that the alarm be adjusted to indicate both high activity (possible agitated state) and low activity (possible illness).

#### Adherence to wearing the smart vest

Many participants were unwilling to wear the smart-vests on the schedule suggested. To enhance participant adherence, the research nurse reminded family caregivers continuously about the need to the wear the smart-vest 24 h a day as often as possible. However, seven participants did not wear the smart-vest at night, preventing monitoring of nighttime activities. The nurse researcher patiently explained to the participants and their caregivers the reason for wearing the smart-vest continuously, including during the night. After hearing the explanation, two participants (S003, S007) agreed to wear the smart-vest at night; however, the other five refused, and did not wear the smart-vest at night during the entire study period (S004, S005, T001, T005, T007).

#### Improper use of alarms

Many of the care receivers (*n* = 8) accidently activated the emergency button (which was silent) and 36 non-emergency calls were generated. To discourage non-emergent calls, the nurse researcher and home care nurse frequently reminded family caregivers to discuss the use of the emergency button with the care receiver and to emphasize that it should only be pressed when needed. Most family caregivers chose not to activate the front door alarm. They explained that they did not think it was necessary because they believed the care receiver would not leave the house by themselves (S003, S005, S007, T001, T002, T003, T005, T006, T007, T008). Two caregivers manually turned the alarm on or off when they felt it was needed (S001, S004). We emphasized that the sensors would not transmit data if the care receiver left the home, however, this did not change the behavior of any of the caregivers.

## Discussion

The STA home-nursing care program developed in this study used smart clothing in the form of a smart-vest with a remote monitoring system to enhance the quality of home nursing care. Smart-home technology transmitted patient data from the smart-vest to home sensors on a daily, 24-hour basis. Data such as posture changes, day and night activities, and toilet frequency were used to establish a normal pattern of the individual’s daily living to help home care nurses and family caregiver become more aware of the individual’s cycles of activity and rest, detect atypical activities, and deal with them as soon as possible.

Two important features were established with our smart care system. First, compared with a smartwatch, the smart-vest not only measures the number of steps, but also calculates changes in the patient’s posture (sitting, standing, lying) and changes in the angle of walking. Our solutions are useful for disabled persons with assistive devices, as opposed to the smartwatch, which cannot be used due to monitor abnormal arm movements, or to analyze a patient’s gait during rehabilitation for hip fracture (left and right swing angle). Second, the smart-vest makes it possible to continuously detect activity and posture over a long period of time.

This study is the first to report the difficulties encountered and the strategies employed to resolve the challenges of implementing a smart-care model for PLWD or recovering from hip fracture in a home setting. We found that the difficulties encountered in setting up this system could be categorized into problems with incompatibility of the home environment, interruptions in data transmission, and inaccuracies in data source.

Smart care has been used to help older adults remain in their homes for aging in place [24, 25] and for family caregivers [26, 27]. Our smart-care program is unique in that its focus was on the assistance it could provide to family caregivers. This included short-term benefits for family caregivers of an older person recovering from hip-fracture surgery as well as long-term benefits for caregivers of PLWD. These findings expand on the difficulties and challenges of providing smart home care for PLWD and older adults recovering from hip-fracture surgery [28, 29]. Similar to other smart-care systems, we encountered problems with battery durability, interruptions during data transmission, and inaccuracies in data sources [27].

Care receivers were resistant to using the smart-vest and caregivers were not diligent about making sure sensors were activated, which was a challenge when attempting to optimize the program for prevention of activities that might endanger the care receiver. The perception of whether a technology is useful has been demonstrated to impact a user’s reluctance to accept technology [30, 31]. This reluctance can be explained by the theoretical framework of the Technology Acceptance Model described by Davis [29], which suggests that two factors determine acceptance: (1) perceived usefulness, and (2) perceived ease of use. Thus, the challenges from the multiple difficulties with the system may have prevented the participants from experiencing or understanding the usefulness of the system, and they certainly did not find the system easy to use. We are confident that resolving these difficulties will enhance acceptance of and adherence to the smart-clothing system in the future, as has been demonstrated for another study [32].

### Limitations

This study had several limitations. First, our small, convenience sample might limit the generalizability of our results. Second, we did not include patients’ and caregivers’ feelings and opinions about the STA home-nursing care model, and we did not evaluate outcomes. This information has been analyzed and reported elsewhere. Lastly, we did not make long-term observations, preventing us from capturing situations that occurred later.

Despite these limitations, the results of this study can serve as a reference for future implementation of smart care in the home setting. We have learned that a stable internet connection with a fixed IP is crucial for setting up a smart-home environment. Therefore, selecting a stable internet carrier is not only critical for the stability of the smart environment, but is also important for reducing the time needed for system installation. Budgeting the cost of setting up new internet connections should be considered for all participants, even those with an existing connection. Finally, quantitative research studies with a large sample size should be conducted to better assess the functionality and usability of the STA program.

## Conclusions

We developed an STA Home Nursing care program for PLWD and those recovering from hip fracture. Our model was characterized by a smart-vest with a remote monitoring system designed to detect behaviors such as getting up at night, staying for prolonged periods in the bathroom, immobility, leaving the house, and daily activity, based on number of steps taken per day. We encountered three main difficulties that were categorized as incompatibility of the system with the home environment, interruptions in data transmissions, and inaccuracies in data sources. There was a wide variety in participants’ home settings and personal situations which were unique to each patient and their caregiver.

Many of the problems encountered were considered prior to conducting the study. However, despite our best efforts, unexpected problems occurred during the installation and implementation of the STA home care system. Theoretically, problems such as battery durability, sensor locations, and robustness of the wireless sensors should have been foreseen. However, real-word situations do not necessarily conform to one’s predictions. We were unable to predict how this system would draw on battery power, the presence of concrete walls in the homes and apartments of the participants, and the large presence of unreliable internet providers, and influence of a caregiver’s attitude towards complying with the protocol. Each of these difficulties had their own unique problems and strategies to resolve them, which can serve as references for others when developing and implementing similar smart-care systems. We believe sharing this information with researchers considering adopting this form of home-care assistance could speed up installation and reduce costs.

## Data Availability

The datasets generated and/or analyzed during the current study are not publicly available due to the principal investigator’s decision to make the data publicly available upon completion of the formal study. To access the data please contact the principal investigator Yea-Ing L Shyu.

## Availability of data and materials

Data will be available upon request. To access the data please contact the principal investigator Yea-Ing L Shyu.

## Abbreviations

**ADC**: analog-to-digital converter
**APP**: mobile application
**BPM**: beats per minute
**ECG**: electrocardiogram
**EMD**: empirical mode decomposition
**GPS**: Global Positioning System
**GC**: G-Sensor for gravitational acceleration
**HMM**: hidden Markov model/modeling
**IP**: Internet protocol
**PLWD**: persons living with dementia
**RSSI**: Received Signal Strength Indicator
**STA**: smart technology-assisted

## References

[1] Uei SL, Tsai CH, Kuo YM (2016). The effect of telehealth systems and satisfaction with health expenditure among patients with metabolic syndrome. Technol Health Care.

[2] Chen MJ, Chen KY, Chiang SJ, Daimon M, Lee JS, Yu EW, Ho CY (2013). A telehealth service model for the treatment of hypertension. J Telemed Telecare.

[3] Chiang LC, Chen WC, Dai YT, Ho YL (2012). The effectiveness of telehealth care on caregiver burden, mastery of stress, and family function among family caregivers of heart failure patients: a quasi-experimental study. Int J Nurs Stud.

[4] Lu JF, Chi MJ, Chen CM (2014). Advocacy of home telehealth care among consumers with chronic conditions. J Clin Nurs.

[5] Amiribesheli M, Benmansour A, Bouchachia A (2015). A review of smart homes in healthcare. J Ambient Intell Hum Comput.

[6] Amiribesheli M, Bouchachia H (2018). A tailored smart home for dementia care. J Ambient Intell Humaniz Comput.

[7] Filipov O (2014). Epidemiology and social burden of the femoral neck fractures. J IMAB.

[8] OdenOdén A, Mccloskey E, Kanis JA, Harvey NC, Johansson H. Burden of high fracture probability worldwide: secular increases 2010–2040. Osteoporos Int. 2015;26. 10.1007/s00198-015-3154-6. 10.1007/s00198-015-3154-6 26018089

[9] Dhanwal DK, Dennison EM, Harvey NC, Cooper C (2011). Epidemiology of hip fracture: worldwide geographic variation. Indian J Orthop.

[10] Prince M, Wimo A, Guerchet M, Ali GC, Wu YT, Prina M, Alzheimer’s Disease International. 2015. World Alzheimer Report 2015. The Global Impact of Dementia: An Analysis of Prevalence, Incidence, Cost and Trends. https://www.alz.co.uk/research/WorldAlzheimerReport2015.pdf. Archived at: https://www.alz.co.uk/research/world-report [accessed 2019-08-16].

[11] World Health Organization. Global Action Plan on the Public Health Response to Dementia 2017–2025. Geneva: World Health Organization.; 2017. ISBN:978-92-4-151348-7.

[12] Sun Y, Lee HJ, Yang SC, Chen TF, Lin KN, Lin CC (2014). A nationwide survey of mild cognitive impairment and dementia, including very mild dementia, in Taiwan. PLoS ONE.

[13] Department of Statistics, Ministry of Interior, Taiwan. 2019. Monthly bulletin of interior statistics: 1.5 Population by age of 0–14, 15–64, 65 + and by 6-year age group. https://www.moi.gov.tw/files/site_stuff/321/1/month/m1-05.xls. Archived at: https://www.moi.gov.tw/files/site_stuff/321/1/month/month_en.html [accessed 2019-08-16] National Development Council, Taiwan. 2018. Population projections for the R.O.C. (Taiwan): 2018 ~ 2065. Archived at: https://www.ndc.gov.tw/en/cp.aspx?n=2E5DCB04C64512CC [accessed 2019-08-16].

[14] Ministry of Health and Welfare. Dementia Prevention and Care Policy and Action Plan 2.0. 2018–2025. 2018. https://www.mohw.gov.tw/cp-139-541-2.html.

[15] Peng LN, Chen WM, Chen CF, Huang CK, Lee WJ, Chen LK (2016). Survival benefits of post-acute care for older patients with hip fractures in Taiwan: a 5-year prospective cohort study. Geriatr Gerontol Int.

[16] Wang J, Lin CC, Yu YS, Yu TC (2015). Wireless sensor-based smart-clothing platform for ECG monitoring. Comput Math Methods Med.

[17] Lin CC, Yang CY, Zhou Z, Wu S (2018). Intelligent health monitoring system based on smart clothing. Int J Distrib Sens Netw.

[18] Lin CC, Liou YS, Zhou Z, Wu S (2019). Intelligent exercise guidance system based on smart clothing. J Med Biol Eng.

[19] Yeh HF, Shao JH, Li CL, Wu CC, Shyu YL (2017). Predictors of postoperative falls in the first and second postoperative years among elderly hip fracture patients. J Clin Nurs.

[20] Bowen GA (2009). Document analysis as a qualitative research method. Qual Res J.

[21] Fereday J, Muir-Cochrane E (2006). Demonstrating rigor using thematic analysis: a hybrid approach of inductive and deductive coding and theme development. Int J Qual Methods.

[22] Saunders B, Sim J, Kingstone T, Baker S, Waterfield J, Bartlam B (2018). Saturation in qualitative research: exploring its conceptualization and operationalization. Qual Quantity.

[23] Lincoln YS, Guba EG (1985). Naturalistic Inquiry.

[24] Orpwood R, Gibbs C, Adlam T, Faulkner R, Meegahawatte D (2005). The design of smart homes for people with dementia - user-interface aspects. Univers Access Inf Soc.

[25] Zhang Q, Li M, Wu Y (2020). Smart home for elderly care: development and challenges in China. BMC Geriatr.

[26] Lindeman DA, Kim KK, Gladstone C, Apesoa-Varano EC. Technology and Caregiving: Emerging Interventions and Directions for Research, *The Gerontologist*, Volume 60, Issue Supplement_1, March 2020, Pages S41–S49, 10.1093/geront/gnz178. 10.1093/geront/gnz178 PMC7019659 32057082

[27] Carnemolla P (2018). Ageing in place and the internet of things– how smart home technologies, the built environment and caregiving intersect. Vis Eng.

[28] Maresova P, Tomsone S, Lameski P, Madureira J, Mendes A, Zdravevski E (2018). Technological solutions for older people with Alzheimer’s disease: review. Curr Alzheimer Res.

[29] Jensen CM, Overgaard S, Wiil UK, Clemensen J (2019). Can tele-health support self-care and empowerment? A qualitative study of hip fracture patients’ experiences with testing an app. SAGE Open Nurs.

[30] Davis FD (1989). Perceived usefulness, perceived ease of use, and user acceptance of information technology. MIS Q.

[31] Davis FD, Bagozzi RP, Warshaw PR (1989). User acceptance of computer technology: a comparison of two theoretical models. Manage Sci.

[32] Hou YJ, Zeng SY, Lin CC, Yang CT, Huang HL, Chen MC, Tsai HH, Liang J, Shyu YL (2022). Smart clothes-assisted home-nursing care program for family caregivers of older persons with dementia and hip fracture: a mixed-methods study. BMC Geriatr.

